# Selected Pathologies of the Male Genital Organs in Bulls, Including Frequency, Significance, and Risk Factors: A Review

**DOI:** 10.3390/ani15192804

**Published:** 2025-09-25

**Authors:** Aleksander F. Butkiewicz, Maciej Zdun, Jędrzej M. Jaśkowski

**Affiliations:** 1 Department of Basic and Preclinical Sciences, Institute of Veterinary Medicine, Faculty of Biological and Veterinary Sciences, Nicolaus Copernicus University, 87-100 Torun, Poland; maciejzdun@umk.pl; 2 Department of Animal Anatomy, Poznan University of Life Sciences, Wojska Polskiego 71C, 60-625 Poznan, Poland; 3 Department of Diagnostics and Clinical Sciences, Institute of Veterinary Medicine, Faculty of Biological and Veterinary Sciences, Nicolaus Copernicus University, 87-100 Torun, Poland; jmjaskowski@umk.pl

**Keywords:** bulls, male genital organs, pathology, frequency, risk factors

## Abstract

**Simple Summary:**

This study provides an overview of the most common genital pathologies in bulls, including their frequency, significance, and risk factors. These pathologies can cause infertility, reduce semen quality, impair copulation, and lead to reluctance to copulate; they also pose a risk of transmitting infectious diseases. This study discusses selected pathologies of the male genital tract, such as orchitis, testicular hypoplasia, testicular degeneration, testicular hydrocele, malignant tumors, spermatocele, penile papillomatosis, penile hematoma, and other diseases that occur with varying frequency in cattle herds. Information is provided on the incidence of these diseases, their causes, and treatment methods. Understanding these issues is important for breeders, herd personnel, and veterinarians. The study also discusses the diagnosis and treatment of these diseases. Measures must be taken to minimize the incidence of genital defects in bulls to improve breeding efficiency and animal health.

**Abstract:**

Bulls can develop various conditions affecting the penis, testicles, and associated structures that reduce fertility, spread disease, and increase costs for farmers. This review synthesizes data from farm fertility examinations and slaughterhouse findings to illustrate the prevalence of these conditions, their causes (including inherited defects, infections, and injuries), their impacts on breeding, and potential interventions. Conditions discussed include orchitis, testicular hypoplasia, testicular degeneration, testicular hydrocele, malignant tumors, spermatocele, penile papillomatosis, and penile hematoma. Lameness and systemic illnesses can also impair a bull’s breeding ability. Regular fertility examinations assessing overall health, testicle size, semen motility, and sperm morphology, coupled with timely medical or surgical treatment, can improve reproductive outcomes. Maintaining national records of birth defects and health histories could aid in preventing the transmission of deleterious traits. Reducing these disorders enhances animal welfare, protects herd fertility and the food supply, and supports the economic viability of cattle producers.

## 1. Introduction

Genital malformations are common defects found in bulls; however, there are no accurate data on the prevalence of these pathologies. Nonetheless, tracking their incidence in herds, identifying potential risk factors, and preventing and eliminating these problems are important for ensuring livestock welfare and increasing economic returns for breeders. To avoid costs and unnecessary animal suffering, a nationwide register of congenital disabilities in bulls should be introduced, including data on each bull’s genetic profile and genealogy, in order to eliminate the potential transmission of defective traits to offspring. Such registries exist, for example, in human medicine [1].

Pathologies of the genital organs in bulls are classified as acquired or congenital lesions. Penile frenulum disorders, congenital penile deformities, stuttering, bacterial and viral lesions, and mechanical trauma are among the most common pathologies of the bull reproductive system [2]. These diseases can cause infertility, reduce semen quality, lead to genitourinary pathologies, and limit the economic potential of breeding. Understanding the causes, symptoms, and treatment of these diseases is important for breeders, herd personnel, veterinarians, and animal technicians [3].

In young bulls, the bull breeding soundness exam (BBSE) is performed based on criteria developed by the Society for Theriogenology [4,5]. The evaluation is conducted at 12–14 months of age and includes a physical examination, measurement of testicular circumference, analysis of semen motility, and assessment of sperm morphology. A bull found to have pathologies is classified as unsatisfactory in terms of reproductive capacity. Typically, estimation of the BBSE is sufficient to identify bulls exhibiting undesirable reproductive traits [6]. The results of such tests are a valuable source of information about defects and diseases of the reproductive system in young bulls. A second source of data is post-mortem examination of bulls of various ages. Under field conditions, a small proportion of pathological changes are sometimes reported to the veterinarian or result in the bull being transferred to a clinic.

This review provides an overview of selected pathologies affecting the male reproductive organs in cattle, with a particular focus on their prevalence (Table 1 and Table 2), diagnostic approaches, therapeutic interventions, and preventive measures.

## 2. Testicular and Epididymal Pathologies

### 2.1. Orchitis

It has been estimated that orchitis affects about 1.94–3.3% of bulls [15]. In young Nellore bulls, the incidence is 2.04% [16]. A more recent pre-slaughter study conducted in 2014–2016 found that orchitis affected 7.1% of bulls [17]. The frequency of orchitis ranges from 2% to 4.4% in post-slaughter studies [11,17]. Initially, testicular inflammation has an acute course before becoming chronic and leading to secondary testicular atrophy [8,18]. Etiological factors are classified as infectious, autoimmune, allergic, and secondary infections resulting from mechanical trauma to the testes [19,20]. More recently, orchitis due to invasion by Trypanosoma evansi (a parasitic Trypanosoma transmitted by certain insects) has been described [20], as well as besnoitiosis, which is endemic in sub-Saharan areas. Cysts of Besnoitia besnoiti have been found in the testes, epididymis, ampullae, blood vessel walls, and striatum, which can interfere with spermatogenesis [21]. In bulls, its invasion can lead to testicular atrophy with azoospermia. Granulomatous orchitis has also been reported following experimental infection of bulls with chlamydophilosis [22].

Unilateral testicular inflammation has been reported in a Holstein Friesian bull infected with Salmonella typhimurium [23]. Treatment of orchitis in bulls requires prompt initiation of targeted antibiotic therapy (except in cases of autoimmune origin), combined with non-steroidal anti-inflammatory drugs, in order to reduce the risk of damage to the spermatogenic epithelium and secondary deterioration of semen quality. In clinical practice, depending on the severity of the lesions, pharmacological treatment, surgical intervention (including unilateral castration), or culling may be applied. Additionally, cold compresses can be used to alleviate inflammatory symptoms. Each case of orchitis requires individual assessment and determination of the cause, as chronic inflammation leads to irreversible damage to the testes and elimination of the bull from breeding [20,24,25].

### 2.2. Hypoplasia Testis

Testicular hypoplasia is a congenital condition, most often of hereditary origin, which may be partial or complete. The only effective method of preventing its spread is the elimination of individuals carrying defective genes. Because the symptoms of hypoplasia can vary greatly, making an accurate diagnosis requires collecting detailed information, including the case history, pedigree data, results of clinical examination of the reproductive organs and semen, and macroscopic and microscopic post-mortem findings [26,27,28].

According to various sources, rates of testicular hypoplasia range from 0.2% of slaughtered bulls [29] to 2.7% and 3.6% of young and older bulls of various meat breeds [30]. It is most common in the Brahmann breed, occurring in 8.6% of bulls [11,30]. Belgian Blue bulls are also prone to hypoplasia and associated poor sperm quality [31]. Testicular hypoplasia has been observed in bulls permanently infected with the BVD virus [32]. Furthermore, it occurs in bulls with a 61 XXY karyotype and in those with XX/XY chimerism [33,34,35].

### 2.3. Degeneratio Testis

Degeneration of the testicles is most often the result of trauma. It can be caused by various thermal or toxic factors, systemic diseases associated with fever, tumors, and nutritional imbalances. Progressive degeneration of the testes and epididymides has been observed in bulls experimentally infected with Trypanosoma vivax (parasitic flagella carried by tse-se flies). Epididymal sperm reserves decreased from 36% to 4% and 0% at 14, 28, and 56 days after infection [36]. Migbaru et al. [11] found that the frequency of testicular degeneration in bulls after slaughter was 8.1%. Degenerative changes can be unilateral or bilateral. The more severe and longer-lasting the testicular injury, the greater the degree of degeneration. Mild trauma to the testes can cause a temporary increase in sperm defects, while prolonged disorders result in progressive degeneration. The testes can partially self-regenerate depending on the extent of damage and the quality of the regeneration process. If the cause is unknown, testicular degeneration is classified as idiopathic. This type of disorder has been observed primarily in older bulls. Degeneration leads to decreased sperm production and lower testosterone levels. Testicular circumference is reduced, and structural changes can be determined by palpation. If the underlying cause is removed, gradual recovery is possible within three to four months [37]. A successful case of treating a bull with bilateral testicular degeneration using gonadoliberin has been described [38].

Ultrasonography can be successfully used for diagnosis. While this method allows for the detection of testicular degeneration, it usually does not enable determination of the underlying cause. In a study by Moran et al. in 2025 [39], ultrasonography was used to diagnose testicular degeneration in Limangus bulls that were culled due to poor reproductive performance.

### 2.4. Hydrocele Testis and Cysts Testes

Testicular hydrocele is very rare. In young bulls, it is recorded in 0.78–1.02% of individuals [15,16]. There are some breed-related differences in the incidence of hydrocele, with cases occurring in about 1% of bulls [40]. Hydrocele has been reported in Holstein Friesian [41], Angus [42], Ongole Guernsey [43], Sahival [44], and other breeds. In calves, it is rare. Lesions can undergo gradual resorption and may affect semen quality. An interesting case of testicular hydrops was described more than a century ago. The initial examination revealed a large hydrops of the left scrotal sac, with the scrotum extending below the ankles.

After thorough examination, fluid aspiration was performed, and a twenty-five per cent solution of tui extract was introduced into the resulting cavity. Ten days later, the scrotum remained a similar size but was much firmer. Surgical intervention was then undertaken, and after removal of the diseased fibrous tissues, the cavity was rinsed with Lugol’s fluid and packed with gauze for 24 h. The next step included irrigation with a 1:2000 dichloride solution. Within four weeks, the scrotal wound had healed, and the scrotum regained its normal size and shape. Eight months later, a tuberculin test was performed in the bull’s herd of origin. The bull with the hydrocele tested positive, suggesting that the hydrocele may have been caused by tuberculosis of the testis or its appendages [45].

**Figure 1:**
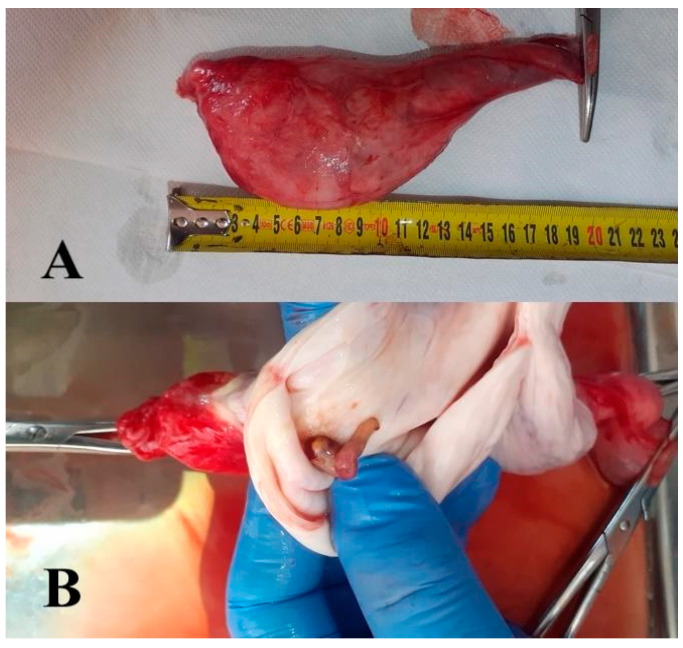
Congenital pseudocyst of the testis (cystis spuria testiculi congenita) in a three-week-old calf. (**A**)—View of the side surface. (**B**)—View after dissection.

Testicular cysts are even rarer. They have been detected within the testicular parenchyma of bulls during ultrasound examinations and are often associated with infertility [46]. Recently, a case of congenital testicular pseudocyst (cystis spuria testiculi congenita) was described in a three-week-old calf [47], representing the largest recorded so far in a bull. The cyst was successfully removed. Castration was performed using the bloody method according to Muir, involving removal of the testis along with the common vaginal tunic (Figure 1).

In cases of hydrocele and testicular cysts, surgical treatment is the method of choice [47,48].

### 2.5. Tumores Neoplasmatices

Neoplastic lesions of the testes are rare in bulls. The most common testicular neoplasm is the Sertole cell tumor. Shorthorn bulls have a relatively high predisposition to this type of cancer [49]. Very rarely, lesions include mesothelioma, hamartoma vascularis, and sarcoma lymphaticum [49]. Only four cases of testicular yolk sac tumors other than seminomas have been reported in bulls. One of these was recently described in a 32-day-old bull, which presented with significant testicular enlargement [50].

The diagnosis of testicular neoplasms in bulls relies primarily on clinical and ultrasonographic examination, whereas definitive confirmation is only possible through histopathology. Due to the exceptional rarity of these lesions, diagnoses are most often made post mortem, while antemortem diagnostic approaches remain poorly developed. Standardized diagnostic criteria for bovine populations are also lacking. From a practical standpoint, testicular neoplasms in bulls are of lesser importance compared with other testicular pathologies, which occur more frequently and have a more pronounced impact on fertility. Nevertheless, they should not be disregarded, as they may result in unilateral or bilateral testicular atrophy, deterioration of semen quality, and ultimately exclusion of bulls from breeding programs. For this reason, they should be considered in the differential diagnosis of testicular lesions [49].

In summary, testicular neoplasms in bulls are rare, and the available knowledge is based almost exclusively on case reports. Population-level studies are lacking, which limits the ability to assess their true prevalence and clinical relevance. Further research is needed to clarify their pathogenesis, improve diagnostic methods, and determine their potential impact on fertility and breeding value in bulls.

### 2.6. Cryptorchidism

Cryptorchidism in bulls is a rare but significant reproductive disorder, documented in post-mortem studies with an incidence of approximately 2.6% [11]. The majority of cases (around 90%) are unilateral, while the remaining 10% are bilateral. Analyses indicate that the left testicle is more commonly retained than the right (69% vs. 31%) [51], and subcutaneous cryptorchidism occurs more frequently than abdominal cryptorchidism [52]. In 66% of cases, the retained testis was located in the inguinal canal [51].

Breed is considered an important risk factor; Polled Hereford and Shorthorn bulls show a higher predisposition to this condition [51]. Similarly, studies have shown that cryptorchidism occurs more frequently in Sokoto Gudali bulls compared to Red Bororo bulls and their crossbreeds [52]. Furthermore, unilateral cryptorchidism is observed more often in bulls with Robertsonian translocation 1/26, suggesting a possible genetic basis [53].

The clinical significance of cryptorchidism includes not only potential fertility problems but also an increased risk of testicular tumors in the retained gonads, as well as challenges in breeding program management. The literature describes a successful laparoscopic method for removal of cryptorchid testes in a standing bull, providing a less invasive and potentially safer alternative to traditional surgery [54].

### 2.7. Epididymitis

Epididymitis in bulls often occurs concurrently with orchitis and is characterized by a similar clinical presentation and potential complications. Clinical signs may include swelling and tenderness of the epididymis, occasional fever, and, in chronic cases, testicular atrophy and reduced semen quality. Kouamo and Eta [13] reported a prevalence of 2–4% in slaughtered bulls, indicating a moderate but significant occurrence in breeding populations. Together with seminal vesiculitis, epididymitis is among the most common causes of accessory gland inflammation in bulls [55].

The etiology of epididymitis is diverse. It is often a consequence of bacterial or viral infections, with Chlamydophila spp. being one documented pathogen responsible for chlamydiosis in cattle [56]. Post-mortem studies have estimated the incidence of epididymitis at 3.7% [17]. Pharmacological treatment is frequently suboptimal, which may result from delayed diagnosis, poor drug penetration into the epididymal tissue, or inappropriate antibiotic selection [55].

The consequences of chronic or inadequately treated epididymitis can be severe. In some cases, permanent damage to the spermatic ducts occurs, leading to impaired sperm transport and reduced fertility in bulls [57]. Therefore, early diagnosis, appropriate treatment, and monitoring of semen quality are essential in breeding practice to minimize reproductive and economic losses.

### 2.8. Spermatocele

Spermatoceles are benign lesions of the epididymis that can disrupt the passage of sperm through its structures and, consequently, affect the reproductive function of bulls [58]. These lesions are most commonly located in the head of the epididymis and result from damage to the seminiferous tubules. The most frequent cause is a congenital developmental defect known as aplasia, characterized by underdevelopment of the organ despite proper formation of its primordium. Unlike the testis, which develops from the genital ridge, the epididymis arises from the Wolffian ducts, making it more susceptible to congenital malformations. These can include aplasia of parts of the epididymis, the vas deferens, and the seminal vesicles, potentially leading to impaired sperm transport and reduced fertility [59,60].

In some cases, spermatoceles arise spontaneously in the head of the epididymis without an apparent cause. They may also develop secondarily due to infections, such as *Brucella abortus* or *Histophilus somni*. However, the literature indicates that most infection-related spermatoceles occur in the body or tail of the epididymis, suggesting differences in the pathogenesis between congenital and acquired lesions [61].

Unilateral spermatoceles generally do not result in complete infertility, provided the contralateral testis is normal and produces semen of adequate quality. Nevertheless, total ejaculate volume is often reduced, which may affect reproductive efficiency. Detection of spermatoceles relies on palpation and ultrasonographic examination, which allows accurate assessment of the lesion’s location, size, and structure [61].

### 2.9. Hernia Scrotalis

Hernia scrotalis is a variant of inguinal hernia in which the hernia sac is located in the scrotum. Scrotal hernias are primarily encountered by veterinarians and pig breeders [62]. Its incidence in cattle is low; according to some researchers, it is 0.87% [15]. Treatment requires surgical intervention, with three main approaches. The first technique is similar to the one commonly used in pigs but unfortunately involves unilateral or bilateral castration by repositioning the hernia sac and then suturing the canal. The second method involves a laparotomy, or opening of the abdominal cavity, and reduction of the hernia sac from the inside by retracting the intestine and suturing the hernia ring in the peritoneal cavity. This technique presents some difficulties, such as suturing “blindly” and the need to operate with one hand. The third method involves an incision over the inguinal canal and repositioning the intestines. The procedure is followed by suturing of the peritoneum [63].

## 3. Penile and Foreskin Pathologies

### 3.1. Fibropapillomatosis

Penile papillomatosis is a relatively common lesion in young bulls [64]. It is caused by the double-stranded DNA bovine papillomavirus (BPV) of the Papillomaviridae family. Infection results in the appearance of characteristic cauliflower-shaped warts in the glans region of the penis [65,66]. The virus is thought to enter the penile skin through minor abrasions that occur during interactions with other males. The virus causes neoplastic growth of fibroblasts but does not cause metastasis. Penile papillomatosis often develops in several bulls from the same production group, but no visible lesions are observed on other parts of the body. Other neoplastic lesions of the penis in cattle are extremely rare and include squamous papilloma, squamous cell carcinoma, and transmissible venereal tumor [67,68,69,70,71].

Although these lesions are benign and non-metastatic, their occurrence can significantly affect the reproductive performance of the herd. Without proper veterinary oversight, untreated cases may facilitate the spread of the virus among young bulls, representing an epidemiological risk. Furthermore, these lesions can cause discomfort and stress in affected animals, potentially impacting overall condition and reproductive behavior.

Papillae on the penile surface make copulation and semen collection difficult. An infected bull serves as a vector for the virus; urination and defecation pose a potential threat to other animals. In addition, semen collected from an infected individual can transmit papillomatosis to the recipient [72]. Stocco dos Santos et al. [73] reported symptoms of BPV following a blood transfusion from an infected individual to a healthy one.

The treatment of choice is surgical resection of the affected parts of the penis using a catheter to precisely locate the external urethral outlet and avoid damage. Penile papillomatosis can recur during periods of active disease. To reduce the risk of recurrence, complete removal of the papillae and the surrounding tissues is recommended. As a prophylactic measure or to reduce the recurrence of lesions, commercial or autologous vaccines against bovine papillomavirus are recommended. Bulls treated for penile fibropapillomatosis should be examined by a veterinarian to assess healing and check for recurrence at least four weeks after treatment before being allowed to breed [2].

### 3.2. Penile Hematoma

**Figure 2:**
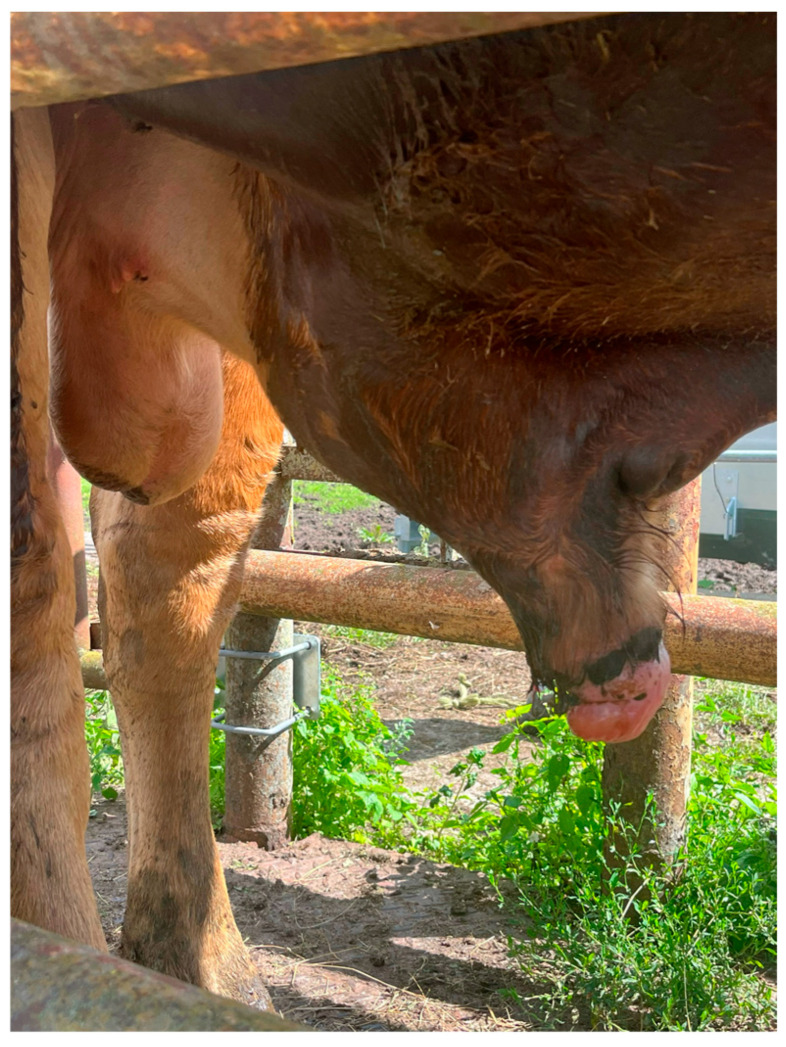
Penile swelling caused by mechanical trauma. The photograph shows eversion of the prepuce due to the inability to retract the penis. The injury occurred during copulation. Conservative management included antibiotic therapy, steroidal anti-inflammatory agents, and anti-edematous medications. Sexual rest was recommended. The treatment resulted in a favorable outcome for the animal. (photo A.F. Butkiewicz).

Penile hematoma is another challenge in veterinary clinical practice. It affects 7–14.9% of bulls at slaughter [13,17]. It usually occurs during the rupture of the penile tunica albuginea, e.g., during mechanical trauma or copulation. Most ruptures are localized on the dorsum of the penis [74]. Injury is usually followed by penile swelling (Figure 2). Two types of therapeutic management are described: conservative treatment, which involves antibiotic therapy and isolation of the bull to prevent contact with other animals, and surgical treatment, which involves removal of the hematoma within seven days of injury [2,75].

### 3.3. Penile Hair Ring

Penile hair ring occurs sporadically [76]. Cases have been reported in young 12–14-month-old bulls kept in the same enclosure [77]. Under such conditions, mutual flopping can occur, leading to the formation of a ring of another individual’s hair or their own around the bull’s copulatory organ. Many cases of penile hair ring resolve spontaneously, but some require surgical intervention [76]. After removal, the ring-shaped lesion may persist for some time. In severe cases, there is a risk of organ necrosis [2,78].

### 3.4. Penile Tuberculosis

Cases of penile tuberculosis have also been described [79,80,81,82]. This zoonosis is caused by Mycobacterium tuberculosis var. bovis [83]. Vielmo et al. (2020) described a bull with a thickened, hard prepuce with a pale inner surface [82]. Multifocal nodules on the preputium ranging from 0.5 to 5 cm in diameter were also observed. This is a unique case, as bovine tuberculosis mainly involves the lungs, the liver, and the tracheobronchial, mediastinal, retropharyngeal, mandibular, mesenteric, and pre-scapular lymph nodes [84]. Penile tuberculosis in cattle most likely results from mating females with uterine tuberculosis [85,86].

### 3.5. Tumors of the Copulatory Organ

Cases of penile fibrosarcoma have been reported [87,88]. This is a particularly unusual type of neoplasm in cattle originating from fibrous connective tissue [89,90,91]. In the case described, the size-differentiated, confined, and non-pigmented tumors were located on the glans penis. They consisted of densely packed and irregular connective tissue with many fibroblasts. Symptoms included painful urination, hematuria, and weight loss [87].

### 3.6. Frenulum Preputii Persistens

The literature on the etiology of this pathology is limited. At present, it is so rare that its significance is considered minor, and some authors [18,92,93] do not attach particular importance to it. Some publications suggest a genetic basis with recessive inheritance, and affected bulls should not be used for breeding [94,95]. Its incidence, assessed post-maturity in bulls, 46% of which were post-maturity, was 0.5% [29].

A persistent frenulum (i.e., retention of the original attachment) is considered pathological in bulls older than eleven months [93]. This condition is treated surgically. The frenulum should be ligated from the temporal and caudal sides to reduce potential bleeding, followed by resection of the foreskin frenulum [2,95].

### 3.7. Phimosis and Paraphimosis

**Figure 3:**
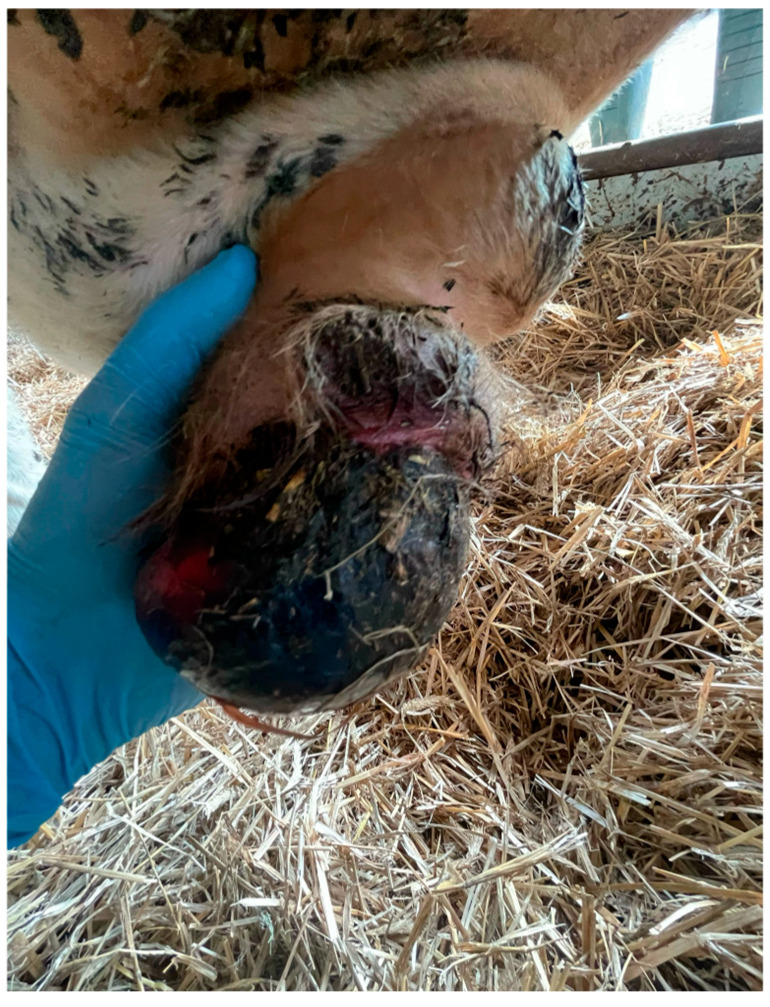
Mechanical injury of the prepuce accompanied by its eversion. The preputial orifice exerts pressure on its external portion. (photo A.F. Butkiewicz).

Phimosis occurs when the penile prepuce cannot be retracted from the glans penis. The condition can be acquired or hereditary. Phimosis can cause pain and discomfort during copulation and bacterial infections of the urethra, preputium, penis, and bladder due to impaired urination [96]. When a stool diagnosis is made, the most common procedure is surgical plication of the external orifice of the foreskin to widen it. The situation becomes more complicated with paraphimosis, a blockage of the prepuce in the retracted position, leading to swelling and impaired blood supply to the glans and the preputium (Figure 3). In most cases, arterial vascularization remains intact for hours or days, although necrosis of the surrounding tissues can occur, sometimes requiring amputation of the penile glans [96,97].

There are three main causes and mechanisms of paraphimosis in bulls. The first is swelling of the preputium, which can result from trauma to the penis or the preputium, as well as diseases that cause abdominal swelling. The second mechanism is damage to penile innervation, which can result from spinal cord injury, mechanical damage, or penile hematoma. The third cause is penile paralysis, which can result from priapism (prolonged non-physiological erection) or the use of acepromazine/phenothiazine as a sedative [98,99,100,101]. Treatment is surgical and involves widening the preputial orifice to free the tissues. A purse-string closure can be used after penile repositioning [96].

### 3.8. Other Penile Defects

Other defects occur occasionally. A corkscrew penis was described in an aged Angus bull [102]. Congenital penile urethral aplasia was described in a 4-day-old bull [103]. Treatment included a permanent perineal urethrostomy and post-operative antibiotic and fluid therapy. Ventral deviation is much less common [2]. Another rare cause of impotence is a congenitally short penis. Diagnosis is based on measuring the length of the penis from the atrium of the preputium to the base of the penis at the blockage of the n. pudendus, with a value above 25 cm considered normal in adults [104]. Young bulls may occasionally have difficulty achieving an erection due to congenital vascular fistulas in the corpora cavernosa. During attempted erection, the free part of the penis may appear bluish, caused by an influx of blood through the subcutaneous vessels. Treatment of such fistulas is usually difficult. Vascular fistulas can also develop as a result of trauma. Attempting to heal the penis after trauma may lead to fistulas that prevent the penis from maintaining normal blood pressure during the erection. Contrast cavernosography can be used to confirm penile vascular malformations, and vascular leaks can be identified using a contrast agent that exits the corpora cavernosa [2,105,106,107].

## 4. Disorders of the Accessory Sex Glands

Pathologies of the accessory genital glands in cattle are described only to a limited extent in the literature, particularly in recent publications. The notable exception is vesicular adenitis. This limited coverage may result from the perception that other conditions are not considered major breeding problems, as well as decreased interest among contemporary researchers, as most available reports are relatively dated. More publications have addressed the physiology of the accessory genital glands and semen collection [108,109,110,111]. In domestic cattle, three principal accessory genital glands are recognized: the prostate, the bulbourethral glands, and the seminal vesicles [112]. Abnormalities of the prostate and the bulbourethral glands in bulls are considered rare [113,114].

In a large-scale study by Bagshaw and Ladds [115] involving 521 bulls, accessory genital glands were collected post-mortem at a slaughterhouse, and numerous abnormalities and pathological changes were identified. In nine bulls, malformations of the mesonephric duct were observed, including aplasia, hypoplasia, or adhesions of the seminal vesicles and ampullae of the vas deferens. Unilateral aplasia of the bulbourethral glands occurred in four cases, and hypoplasia was found in four older bulls.

Melanosis (the presence of black melanin spots) was diagnosed in five animals. Cysts of the seminal vesicles exceeding 4 mm in diameter were found in eight bulls; in five cases, the glands were macroscopically normal, and in three they were enlarged; histological examination frequently revealed concurrent inflammation. Prostate cysts measuring 0.5 cm in diameter were recorded in one bull, and bulbourethral gland cysts >0.5 cm were found in six bulls, one of which exhibited inflammatory changes.

Vesiculitis was diagnosed in 47 bulls (9.0%), of which 14 cases (2.7%) would have been detectable via rectum examination, underscoring the higher sensitivity of post-mortem evaluation. Of these cases, 17 were chronic interstitial (unilateral, *n* = 8; bilateral, *n* = 9), frequently accompanied by adhesions, abscesses (*n* = 4), cysts (*n* = 4), calculi (*n* = 5), and concurrent ampullitis (*n* = 11). The remaining 30 cases were degenerative (unilateral, *n* = 13; bilateral, *n* = 17), sometimes associated with abscesses (*n* = 1), cysts (*n* = 2), calculi (*n* = 4), and ampullitis (*n* = 6).

Ampullitis was detected in 20 bulls, including 10 with concurrent vesiculitis; pathological changes included glandular enlargement, increased firmness, and occasional fibrosis. Inflammation of the bulbourethral glands was recorded in one bull, characterized by plasma cell infiltration.

Cysts within the urogenital fold (cystic uterus masculinus) were found in 34 bulls (6.5%), most frequently in the Brahman breed (18%). The study concluded that no single factor or pathology could be definitively linked to infertility in the examined bulls [16].

In another study of the bulbourethral glands in Bos indicus cattle and crossbreeds, adenitis was identified in four bulls (1.2%). In two cases, unilateral chronic interstitial inflammation was present, one of which was associated with degenerative vesiculitis. The remaining cases were bilateral; in one, a foreign body was located in the duct of the left gland, and, in another, multiple calculi and chronic, active diffuse inflammation were observed. The authors hypothesized that the foreign body may have migrated proximally from the prepuce or distal urethra. The impact of these lesions on fertility remains uncertain [116].

Congenital abnormalities of the accessory genital glands may predispose bulls to inflammatory conditions, particularly in the presence of ductal outlet disorders at the colliculus seminalis. Anomalies, such as segmental aplasia or hypoplasia of the mesonephric ducts, are generally not associated with inflammation, although hypoplasia has occasionally led to inflammatory changes. Early detection of defects—ideally, during puberty—is important in the selection of breeding bulls [2].

Vesiculitis occurs in 1–10% of bulls and can significantly impair fertility. Causative agents include bacteria, chlamydia, mycoplasmas, and ureaplasmas; the most commonly isolated pathogens are Trueperella pyogenes, Histophilus somni, and Brucella abortus (in regions where brucellosis persists). Routes of infection include ascending, descending, local, and hematogenous spread. Congenital defects of the colliculus seminalis and umbilical abscesses in calves increase disease risk [2].

Treatment options include antibiotic therapy, with tulathromycin reported as the most effective agent, administered locally or systemically. Other options include chemical ablation using formalin and surgical excision of the glands in chronic cases [2,117]. Preventive measures involve sound breeding practices, balanced nutrition, deworming, and genetic monitoring, as the pathogenesis is still not fully understood. Tilmicosin may also be effective [118]. Direct intraglandular injection of ceftiofur or penicillin is possible but technically challenging [119].

Recent experimental approaches have included the injection of stem cells into the seminal vesicles. Allogeneic mesenchymal stem cells (MSCs), administered directly into the vesicles of bulls with chronic vesiculitis, improved semen quality and promoted glandular regeneration. In a study of 12 bulls, MSC therapy resulted in increased sperm motility, greater semen production, and complete elimination of leukocytes from semen, confirming both the efficacy and safety of this method [120].

A surgical technique for removal of the seminal vesicles has also been developed; however, its success is not guaranteed and the procedure should be reserved as a last resort [121].

## 5. Sterilitas Secundaria

There are a number of pathologies that are unrelated to the reproductive system but significantly affect a bull’s willingness to copulate and the quality of semen. Such pathologies include laminitis, fractures, interdigital hyperplasia, sole ulcer, arthritis, spinal degeneration and other diseases that affect the animal’s welfare and perception of potential discomfort or pain [122,123,124,125,126,127,128,129,130,131,132,133,134,135,136,137,138,139].

The introduction of registries for congenital genital defects in bulls is important for animal welfare and offers potential economic benefits for breeders. Although these defects are common, accurate data on their incidence are lacking. Pathologies of the bull’s reproductive system, such as orchitis, testicular hypoplasia, testicular degeneration, and testicular hydrocele, can lead to infertility, reduced semen quality, and decreased economic potential in breeding. The Bull Breeding Soundness Evaluation, which includes physical examination, testicular circumference measurement, assessment of semen motility, and sperm morphology analysis, is crucial for evaluating the reproductive condition of bulls. The results provide valuable information on defects and diseases of the reproductive system in young bulls. Proper identification of these pathologies, as well as understanding their causes, symptoms, and treatment methods, is essential for breeders, herd personnel, veterinarians, and animal technicians.

## 6. Conclusions

In summary, comprehensive understanding, early detection, and effective treatment of congenital and acquired disorders of the reproductive organs in bulls are essential for ensuring animal welfare, optimizing reproductive performance, and minimizing economic losses in cattle breeding. The diversity of pathologies within the reproductive system highlights the need for systematic veterinary assessment, precise diagnostics, and timely interventions.

Future research should focus on further exploring the genetic and molecular bases of these disorders, developing minimally invasive therapeutic methods, and investigating preventive measures, such as vaccinations against infectious etiologies. The establishment of national and international registries of reproductive defects would provide valuable epidemiological data, support selective breeding programs, and improve herd management strategies. Integrating such registries with modern breeding technologies could enable predictive monitoring and early intervention, further enhancing reproductive outcomes.

Moreover, modern cattle breeding researchers should aim to utilize artificial intelligence; for instance, by developing programs that are capable of predicting the occurrence of congenital defects. Ultimately, a multidisciplinary approach combining veterinary medicine, genetics, advanced diagnostics, and herd management will be key to improving health and reproduction. These initiatives may contribute to increasing breeding efficiency, reducing animal suffering, and striving for excellence in genetic selection.

## Figures and Tables

**Table 1:** Selected pathologies of the male genital organs in bulls.

Pathology	Etiological Factor	Type of Defect	Treatment	Estimated Frequency [%]
Orchitis	Infectious, autoimmune, allergic, mechanical trauma	Acquired	Antibiotic therapy	1.94–7.1%
Hypoplasia testis	BVD virus, genetic mutation	Congenital. acquired	Cull, vaccines	0.2–3.6%
Degeneratio testis	Trauma, tumours, deficiency of nutrients, Trypanosoma vivax, idiopathic	Acquired	Cull, GnRH	8.1%
Hydrocele testis	Trauma, tumours, orchitis, epididymitis	Acquired	Surgical, self-treatment	1%
Cryptochidism	Genetic mutation	Congenital	Surgical	2.6%
Epididymitis	Infectious, autoimmune, allergic, mechanical trauma, chlamydophilia,	Acquired	Antibiotic therapy, cull	3.7%
Hernia scrotalis	?	Congenital. acquired	Surgical	0.87%
Penile papillomatosis	BPV virus	Acquired	Surgical, vaccines	?
Penile hematoma	Trauma	Acquired	Antibiotic therapy, surgical	7–14.9%
Penile hair ring	Trauma	Acquired	Self-treatment, surgical	?
Frenulum preputii persistens	Genetic mutation	Congenital	Surgical	0.5%
Phimosis and paraphimosis	Trauma, genetic mutation, paralysis	Congenital. acquired	Self-treatment, surgical	?

**Table 2:** Prevalence of selected reproductive system pathologies in bulls across different regions, breeds, and studies.

Pathology	Country/Region	Breed	Frequency (%)	Authors
Hypoplasia	Cameroon	Local breeds	5.8	Kouamo & Nyonga 2022 [7]
	Algeria	Local breeds	0.66	Bousmaha & Khoudja 2012 [8]
	Mexico	Brahman	3.45	Silva et al., 2008 [9]
	Mexico	Nelore	1.87	Silva et al., 2008 [9]
	Mexico	Brown Swiss	3.1	Silva et al., 2008 [9]
	Australia	Santa Gertrudis	1.4	McGowan et al., 2002 [10]
	Australia	Brown Swiss	3.1	McGowan et al., 2002 [10]
	Ethiopia	Local breeds	3.6	Migbaru et al., 2014 [11]
	Ethiopia	Local breeds	18.8	Gemeda 2017 [12]
	Cameroon	Local breeds	8.9	Kouamo & Eta 2021 [13]
	Turkey	Mixed	1.67	Uyar et al., 2019 [14]
Testicular degeneration	Cameroon	Local breeds	2.5	Kouamo & Nyonga 2022 [7]
	Ethiopia	Local breeds	6.5	Gemeda 2017 [12]
	Ethiopia	Local breeds	8.1	Migbaru et al., 2014 [11]
	Cameroon	Local breeds	1.0	Kouamo & Eta 2021 [13]
Cryptorchidism	Cameroon	Local breeds	1.3	Kouamo & Nyonga 2022 [7]
	Ethiopia	Local breeds	1.0	Gemeda 2017 [12]
	Mexico	Brahman	0.49	Silva et al., 2008 [9]
	Mexico	Brown Swiss	0.92	Silva et al., 2008 [9]
	Ethiopia	Local breeds	2.6	Migbaru et al., 2014 [11]
	Turkey	Mixed	0.44	Uyar et al., 2019 [14]
Hematoma (testicles/penile)	Cameroon	Local breeds	1.9	Kouamo & Nyonga 2022 [7]
	Cameroon	Local breeds	14.9	Kouamo & Eta 2021 [13]
	Ethiopia	Local breeds	9.0	Gemeda 2017 [12]
	Ethiopia	Local breeds	2.1	Migbaru et al., 2014 [11]
Orchitis	Cameroon	Local breeds	0.5	Kouamo & Nyonga 2022 [7]
	Cameroon	Local breeds	7.0	Kouamo & Eta 2021 [13]
	Ethiopia	Local breeds	4.4	Migbaru et al., 2014 [11]
	Turkey	Mixed	1.23	Uyar et al., 2019 [14]
Epididymitis	Cameroon	Local breeds	0.2	Kouamo & Nyonga 2022 [7]
	Cameroon	Local breeds	2.0	Kouamo & Eta 2021 [13]
	Ethiopia	Local breeds	3.4	Migbaru et al., 2014 [11]
	Australia	Santa Gertrudis	3.0	McGowan et al., 2002 [10]
	Ethiopia	Local breeds	2.5	Gemeda 2017 [12]

## Data Availability

The data presented in this study are available on request from the corresponding author.
